# dl-Tyrosinium chloride dihydrate

**DOI:** 10.1107/S1600536812043899

**Published:** 2012-10-27

**Authors:** Fatiha Guenifa, Lamia Bendjeddou, Aouatef Cherouana, Slimane Dahaoui, Claude Lecomte

**Affiliations:** a Unité de Recherche Chimie de l’Environnement et Moleculaire Structurale, (CHEMS), Faculté des Sciences Exactes, Campus Chaabet Ersas, Université Mentouri de Constantine, 25000 Constantine, Algeria; b Cristallographie, Résonance Magnétique et Modélisation (CRM2), Université Henri Poincaré, Nancy 1, Faculté des Sciences, BP 70239, 54506 Vandoeuvre lès Nancy CEDEX, France

## Abstract

In the title compound, C_9_H_12_NO_3_^+^·Cl^−^·2H_2_O, the cation has a protonated amino group resulting from proton transfer from chloridric acid. The structure displays double layers parallel to the [010] direction held together by N—H⋯O, N—H⋯Cl, O—H⋯O and O—H⋯Cl hydrogen bonds. These layers are stacked along the *c* axis at *b* = 1/2; within each layer, the tyrosinium cations are arranged in an alternating head-to-tail sequence, forming inversion dimers [*R*_2_^2^(10) motif]. The water mol­ecules allow for the construction of a three-dimensional hydrogen-bonded network formed by centrosymmetric *R*_6_^6^(28) and *R*_8_^8^(34) motifs.

## Related literature

For other examples of organic salts of amino acids, see: Zeghouan *et al.* (2012 ▶); Guenifa *et al.* (2009 ▶). For the structure of bis­(l-tyrosinium) sulfate monohydrate, see: Sridhar *et al.* (2002 ▶). For other examples of amino acids with non-polar side chains, see: Torii & Iitaka (1973 ▶); Harding & Long (1968 ▶). For graph-set notation, see: Bernstein *et al.* (1995 ▶).
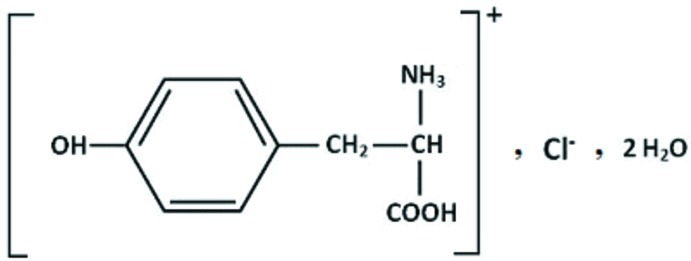


## Experimental

### 

#### Crystal data

C_9_H_12_NO_3_^+^·Cl^−^·2H_2_O
*M* *_r_* = 253.68
Triclinic, 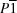

*a* = 5.3330 (2) Å
*b* = 10.9634 (5) Å
*c* = 11.2500 (4) Å
α = 113.642 (4)°
β = 94.359 (3)°
γ = 98.465 (3)°
*V* = 589.34 (5) Å^3^
*Z* = 2
Mo *K*α radiation
μ = 0.33 mm^−1^
*T* = 100 K
0.3 × 0.03 × 0.02 mm

#### Data collection

Oxford Diffraction Xcalibur Sapphire CCD diffractometer
12044 measured reflections
3445 independent reflections
2780 reflections with *I* > 2σ(*I*)
*R*_int_ = 0.034

#### Refinement

*R*[*F* ^2^ > 2σ(*F* ^2^)] = 0.032
*wR*(*F* ^2^) = 0.084
*S* = 1.02
3445 reflections
172 parameters
11 restraints
H atoms treated by a mixture of independent and constrained refinement
Δρ_max_ = 0.43 e Å^−3^
Δρ_min_ = −0.30 e Å^−3^

### 

Data collection: *CrysAlis CCD* (Oxford Diffraction, 2008 ▶); cell refinement: *CrysAlis CCD*; data reduction: *CrysAlis RED* (Oxford Diffraction, 2008 ▶); program(s) used to solve structure: *SIR92* (Altomare *et al.*, 1993 ▶); program(s) used to refine structure: *SHELXL97* (Sheldrick, 2008 ▶); molecular graphics: *ORTEP-3* (Farrugia, 1997 ▶); software used to prepare material for publication: *WinGX* (Farrugia, 1999 ▶), *PARST97* (Nardelli, 1995 ▶), *Mercury* (Macrae *et al.*, 2006 ▶) and *POVRay* (Persistence of Vision Team, 2004 ▶)’.

## Supplementary Material

Crystal structure: contains datablock(s) global, I. DOI: 10.1107/S1600536812043899/nk2187sup1.cif

Structure factors: contains datablock(s) I. DOI: 10.1107/S1600536812043899/nk2187Isup2.hkl

Additional supplementary materials:  crystallographic information; 3D view; checkCIF report

## Figures and Tables

**Table 1:** Hydrogen-bond geometry (Å, °)

*D*—H⋯*A*	*D*—H	H⋯*A*	*D*⋯*A*	*D*—H⋯*A*
N1—H1*N*⋯Cl1	0.90 (1)	2.36 (1)	3.2326 (12)	162 (1)
N1—H2*N*⋯Cl1^i^	0.92 (1)	2.44 (1)	3.2872 (12)	154 (1)
N1—H2*N*⋯O3^i^	0.92 (1)	2.40 (2)	2.9574 (15)	119 (1)
N1—H3*N*⋯Cl1^ii^	0.90 (1)	2.32 (1)	3.2151 (12)	176 (1)
O1—H1⋯Cl1^iii^	0.84 (2)	2.36 (2)	3.1858 (11)	169 (1)
O2—H2⋯O2*W* ^i^	0.89 (1)	1.64 (1)	2.5319 (15)	174 (2)
O1*W*—H11*W*⋯O1^iv^	0.84 (1)	2.10 (1)	2.9044 (13)	162 (2)
O1*W*—H12*W*⋯Cl1^v^	0.85 (1)	2.33 (1)	3.1784 (11)	172 (2)
O2*W*—H21*W*⋯O1*W* ^i^	0.83 (1)	1.91 (1)	2.7429 (14)	173 (2)
O2*W*—H22*W*⋯O1*W* ^vi^	0.85 (2)	2.02 (2)	2.8318 (15)	161 (2)

Symmetry codes: (i) 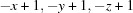; (ii) 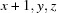; (iii) 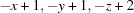; (iv) 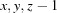; (v) 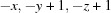; (vi) 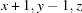.

**Fig. 1.**
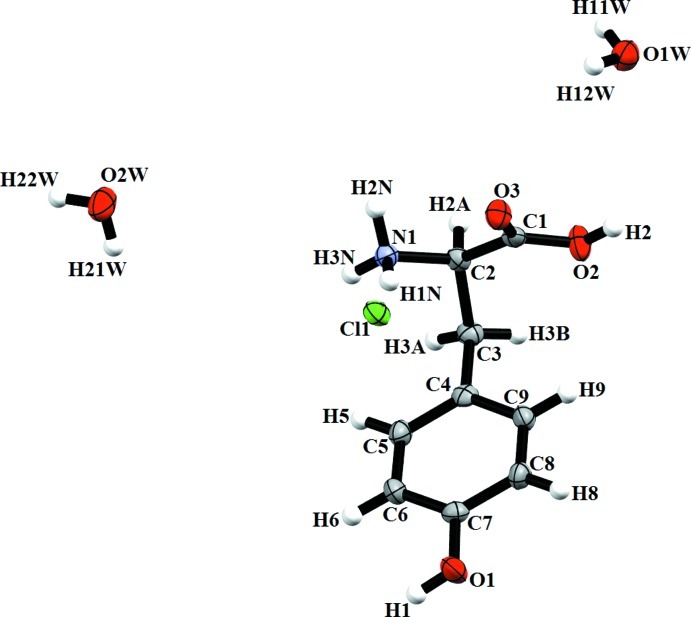
The asymmetric unit of (I) (Fig.1), showing the crystallographic numbering scheme. Displacement ellipsoids are drawn at the 50% probability level and H atoms are shown as spheres of arbitrary radii. Hydrogen bonds are shown as dashed lines.

**Fig. 2.**
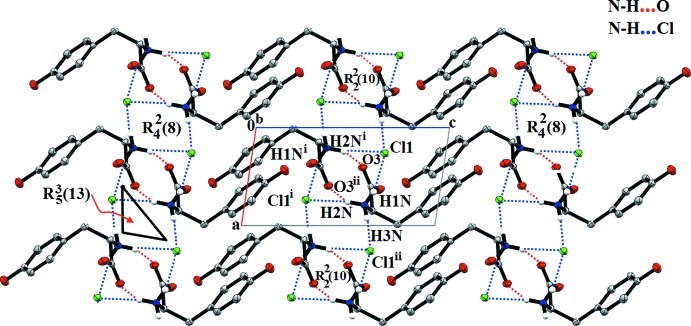
Part of the crystal structures, showing the formation of dimers via N—H···O hydrogen bonds, and the aggregation of R22(10), R24(8) and R35(13) motifs [Symmetry codes: (i) -x + 1, -y + 1, -z + 1; (ii) x + 1, y, z]. For the sake of clarity, the water molecules in (I), and H atoms not involved in hydrogen bonding have been omitted. Only atoms involved in hydrogen bonding are labelled.

**Fig. 3.**
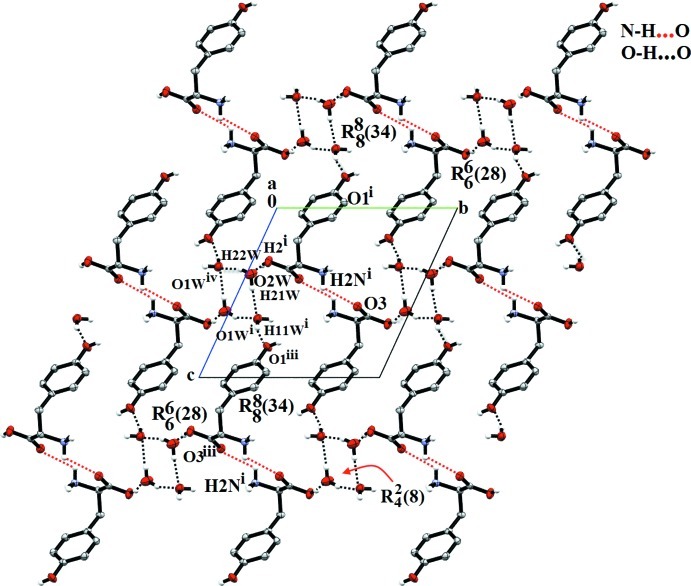
Packing view of (I) showing the aggregation of R24(8), R66(28) and R88(34) hydrogen-bonding motifs. [Symmetry codes:(i) -x + 1, -y + 1, -z + 1; (iii) -x + 1, -y + 1, -z + 2]. For the sake of clarity, the chloride anions, and H atoms not involved in hydrogen bonding have been omitted. Only atoms involved in hydrogen bonding are labelled.

## supplementary crystallographic information

### Comment

We report the crystal structure of DL-tyrosinium chloride dihydrate (I), as part of our research with organic salts of amino acids (Zeghouan *et al.*, 2012; Guenifa *et al.*, 2009).

The asymmetric unit of (I) contains a tyrosinium cation, chloride anion and two water molecules (Fig.1). As expected, tyrosine form protonated units in (I) with the transfer of an H atom from chloridric acid. A similar situation is observed in bis(*L*-tyrosinium) sulfate monohydrate, (Sridhar *et al.*, 2002).

In the crystal structure of (I), the ions are connected into a three-dimensional hydrogen-bonded network *via* N—H···O, N—H···Cl, O—H···O and O—H···Cl hydrogen bonds (Table 1). The tyrosinium cations are held together by N—H···O hydrogen bonds, forming a centrosymmetric dimer (*R*^2^_2_(10) motif; Bernstein *et al.*, 1995) centred at (1/2, 1/2, 1/2). This centrosymmetric dimer is further connected along [100] direction to either side of the chloride anions by N—H···Cl hydrogen bonds [*R*^2^_4_(8) and *R*^3^_5_(13) motifs] (Fig. 2). The aggregation of the rings motifs results in an overall two-dimensional hydrogen-bonded network.

The water molecules, which plays a dual role as both donor and acceptor in hydrogen bonding interactions, generating the centrosymmetric hydrogen-bonded (*R*^2^_4_(8) motif) *via* O2w—H21w···O1w^(i)^ and O2w—H22w···O1w^(vi)^ (Fig. 3), and are involved in two centred hydrogen bonding with the cations to produce a centrosymmetric *R*^6^_6_(28) and *R*^8^_8_(34) motifs, thus completing the three-dimensional hydrogen-bonded network. The structures of many amino acids with non-polar side chains have the arrangement of a double layers of carboxyl and amino groups held together by hydrogen bonds (Torii & Iitaka, 1973; Harding & Long, 1968).

The molecule packing of (I), consists of double layers stacked along the *c* axis, at b = 1/2, where in each layer the tyrosinium cations are arranged with alternating head-to-tail sequence.

### Experimental

The compound was obtained as colourless crystals with melting points of 370°, after few days, by slow evaporation from an aqueous solution of tyrosine and chloridric acid in stoechiometric ratio of 1:1.

### Refinement

The methine, methylene, and aromatic H atoms were placed at calculated positions respectively with C—H fixed at 0.98 Å (AFIX 13), 0.97 Å (AFIX 23), and C—H = 0.93 Å (Afix 43). All H atom attached to N or O were initially located by difference maps with restraint of the N—H bond length to 0.90 (2) Å (*DFIX*), and U fixed to be 1.2 times that of the N1; and O—H bond length to 0.85 (2) Å (*DFIX*) for hydroxyl group and 0.85 (1) Å (*DFIX*) for water molecule with H···H = 1.39 (2) and U fixed to be 1.5 times that of the o1, O2, o1w and o2w.

### Crystal data

**Table:**

C_9_H_12_NO_3_^+^·Cl^−^·2H_2_O	*Z* = 2
*M**_r_* = 253.68	*F*(000) = 268
Triclinic, *P*1	*D*_x_ = 1.43 Mg m^−^^3^
Hall symbol: -P 1	Mo *K*α radiation, λ = 0.71073 Å
*a* = 5.3330 (2) Å	Cell parameters from 12044 reflections
*b* = 10.9634 (5) Å	θ = 3.4–30.0°
*c* = 11.2500 (4) Å	µ = 0.33 mm^−^^1^
α = 113.642 (4)°	*T* = 100 K
β = 94.359 (3)°	Needle, colourless
γ = 98.465 (3)°	0.3 × 0.03 × 0.02 mm
*V* = 589.34 (5) Å^3^	

### Data collection

**Table:**

Oxford Diffraction Xcalibur Sapphire CCD diffractometer	2780 reflections with *I* > 2σ(*I*)
Radiation source: fine-focus sealed tube	*R*_int_ = 0.034
Graphite monochromator	θ_max_ = 30.0°, θ_min_ = 3.4°
ω scans	*h* = −7→7
12044 measured reflections	*k* = −15→15
3445 independent reflections	*l* = −15→15

### Refinement

**Table:**

Refinement on *F*^2^	11 restraints
Least-squares matrix: full	H atoms treated by a mixture of independent and constrained refinement
*R*[*F*^2^ > 2σ(*F*^2^)] = 0.032	*w* = 1/[σ^2^(*F*_o_^2^) + (0.0483*P*)^2^] where *P* = (*F*_o_^2^ + 2*F*_c_^2^)/3
*wR*(*F*^2^) = 0.084	(Δ/σ)_max_ < 0.001
*S* = 1.02	Δρ_max_ = 0.43 e Å^−^^3^
3445 reflections	Δρ_min_ = −0.30 e Å^−^^3^
172 parameters	

### Special details

**Table:**

Geometry. Bond distances, angles *etc*. have been calculated using the rounded fractional coordinates. All su's are estimated from the variances of the (full) variance-covariance matrix. The cell e.s.d.'s are taken into account in the estimation of distances, angles and torsion angles
Refinement. Refinement of *F*^2^ against ALL reflections. The weighted *R*-factor *wR* and goodness of fit *S* are based on *F*^2^, conventional *R*-factors *R* are based on *F*, with *F* set to zero for negative *F*^2^. The threshold expression of *F*^2^ > σ(*F*^2^) is used only for calculating *R*-factors(gt) *etc*. and is not relevant to the choice of reflections for refinement. *R*-factors based on *F*^2^ are statistically about twice as large as those based on *F*, and *R*- factors based on ALL data will be even larger.

### Fractional atomic coordinates and isotropic or equivalent isotropic displacement parameters (Å^2^)

**Table:**

	*x*	*y*	*z*	*U*_iso_*/*U*_eq_	
Cl1	0.26233 (5)	0.45517 (3)	0.68584 (3)	0.01279 (8)	
O2	0.63481 (17)	0.90937 (9)	0.68369 (9)	0.0169 (2)	
O1	0.43456 (17)	0.69839 (9)	1.18096 (9)	0.0162 (2)	
O1W	0.07015 (18)	0.82478 (9)	0.34641 (9)	0.0165 (2)	
H12W	−0.008 (3)	0.7497 (11)	0.3437 (15)	0.025*	
H11W	0.177 (2)	0.8037 (15)	0.2947 (14)	0.025*	
O3	0.40595 (16)	0.69671 (9)	0.58151 (9)	0.01381 (19)	
O2W	0.7642 (2)	0.02475 (10)	0.39042 (10)	0.0255 (2)	
H22W	0.832 (3)	−0.0447 (13)	0.3609 (15)	0.038*	
H21W	0.807 (3)	0.0653 (16)	0.4715 (9)	0.038*	
N1	0.7923 (2)	0.58230 (10)	0.62048 (11)	0.0113 (2)	
C5	0.8819 (2)	0.66936 (12)	0.94214 (12)	0.0130 (2)	
H5	1.0026	0.6179	0.9052	0.016*	
C6	0.7489 (2)	0.64160 (12)	1.03283 (12)	0.0135 (2)	
H6	0.7796	0.572	1.0558	0.016*	
C1	0.6041 (2)	0.77693 (12)	0.63928 (12)	0.0107 (2)	
C7	0.5693 (2)	0.71907 (12)	1.08898 (12)	0.0116 (2)	
C3	0.9865 (2)	0.80241 (12)	0.80762 (12)	0.0121 (2)	
H3A	1.1505	0.7748	0.8117	0.015*	
H3B	1.0206	0.8997	0.8337	0.015*	
C9	0.6553 (2)	0.84770 (12)	0.96215 (12)	0.0122 (2)	
H9	0.6224	0.9165	0.9384	0.015*	
C2	0.8520 (2)	0.73226 (12)	0.66507 (12)	0.0108 (2)	
H2A	0.9708	0.7523	0.6101	0.013*	
C8	0.5211 (2)	0.82194 (12)	1.05327 (12)	0.0128 (2)	
H8	0.3999	0.8731	1.0901	0.015*	
C4	0.8385 (2)	0.77273 (12)	0.90528 (11)	0.0109 (2)	
H3N	0.925 (2)	0.5502 (14)	0.6426 (14)	0.013*	
H2N	0.758 (3)	0.5428 (14)	0.5303 (12)	0.013*	
H1N	0.657 (2)	0.5615 (14)	0.6569 (13)	0.013*	
H1	0.500 (3)	0.6478 (14)	1.2082 (15)	0.016*	
H2	0.494 (2)	0.9367 (14)	0.6636 (14)	0.016*	

### Atomic displacement parameters (Å^2^)

**Table:**

	*U*^11^	*U*^22^	*U*^33^	*U*^12^	*U*^13^	*U*^23^
Cl1	0.01191 (14)	0.01584 (14)	0.01394 (15)	0.00480 (11)	0.00313 (10)	0.00869 (11)
O2	0.0146 (4)	0.0119 (4)	0.0229 (5)	0.0042 (4)	−0.0028 (4)	0.0065 (4)
O1	0.0190 (5)	0.0204 (5)	0.0170 (5)	0.0102 (4)	0.0075 (4)	0.0128 (4)
O1W	0.0177 (5)	0.0145 (4)	0.0176 (5)	0.0041 (4)	0.0055 (4)	0.0061 (4)
O3	0.0111 (4)	0.0137 (4)	0.0161 (5)	0.0032 (3)	0.0010 (3)	0.0055 (4)
O2W	0.0305 (6)	0.0247 (5)	0.0180 (5)	0.0184 (5)	−0.0029 (4)	0.0022 (4)
N1	0.0103 (5)	0.0130 (5)	0.0108 (5)	0.0045 (4)	0.0012 (4)	0.0045 (4)
C5	0.0127 (5)	0.0140 (6)	0.0121 (6)	0.0066 (5)	0.0013 (4)	0.0041 (5)
C6	0.0161 (6)	0.0136 (6)	0.0128 (6)	0.0066 (5)	0.0014 (5)	0.0064 (5)
C1	0.0127 (5)	0.0131 (5)	0.0084 (6)	0.0045 (5)	0.0031 (4)	0.0056 (4)
C7	0.0113 (5)	0.0134 (5)	0.0091 (6)	0.0018 (4)	0.0006 (4)	0.0043 (4)
C3	0.0105 (5)	0.0136 (6)	0.0115 (6)	0.0020 (5)	−0.0004 (4)	0.0050 (5)
C9	0.0140 (6)	0.0106 (5)	0.0126 (6)	0.0037 (5)	0.0001 (5)	0.0053 (5)
C2	0.0103 (5)	0.0120 (5)	0.0113 (6)	0.0026 (4)	0.0016 (4)	0.0058 (5)
C8	0.0133 (6)	0.0122 (5)	0.0131 (6)	0.0059 (5)	0.0024 (5)	0.0041 (5)
C4	0.0095 (5)	0.0115 (5)	0.0087 (6)	0.0002 (4)	−0.0021 (4)	0.0025 (4)

### Geometric parameters (Å, º)

**Table:**

O1—C7	1.3754 (16)	C3—C4	1.5096 (17)
O2—C1	1.3117 (17)	C4—C9	1.3961 (16)
O3—C1	1.2150 (15)	C4—C5	1.3962 (19)
O1—H1	0.837 (17)	C5—C6	1.3900 (18)
O2—H2	0.894 (12)	C6—C7	1.3925 (17)
O1W—H11W	0.835 (12)	C7—C8	1.390 (2)
O1W—H12W	0.854 (14)	C8—C9	1.3882 (18)
O2W—H21W	0.834 (9)	C2—H2A	0.9800
O2W—H22W	0.847 (16)	C3—H3A	0.9700
N1—C2	1.4887 (18)	C3—H3B	0.9700
N1—H2N	0.920 (12)	C5—H5	0.9300
N1—H1N	0.901 (13)	C6—H6	0.9300
N1—H3N	0.896 (13)	C8—H8	0.9300
C1—C2	1.5225 (16)	C9—H9	0.9300
C2—C3	1.5334 (17)		
			
C7—O1—H1	109.9 (11)	O1—C7—C8	117.48 (10)
C1—O2—H2	112.8 (10)	C6—C7—C8	120.27 (11)
H11W—O1W—H12W	105.2 (16)	O1—C7—C6	122.25 (12)
H21W—O2W—H22W	109.8 (16)	C7—C8—C9	119.46 (11)
H1N—N1—H2N	111.9 (13)	C4—C9—C8	121.56 (13)
C2—N1—H2N	108.2 (10)	N1—C2—H2A	108.00
C2—N1—H3N	112.5 (10)	C3—C2—H2A	108.00
H1N—N1—H3N	109.5 (13)	C1—C2—H2A	108.00
C2—N1—H1N	108.3 (10)	C2—C3—H3B	109.00
H2N—N1—H3N	106.6 (14)	C4—C3—H3A	109.00
O2—C1—O3	125.48 (11)	H3A—C3—H3B	108.00
O2—C1—C2	111.91 (10)	C4—C3—H3B	109.00
O3—C1—C2	122.60 (12)	C2—C3—H3A	109.00
N1—C2—C3	110.77 (11)	C4—C5—H5	119.00
C1—C2—C3	115.02 (10)	C6—C5—H5	119.00
N1—C2—C1	107.62 (10)	C7—C6—H6	120.00
C2—C3—C4	114.94 (10)	C5—C6—H6	120.00
C3—C4—C9	121.35 (12)	C7—C8—H8	120.00
C3—C4—C5	120.83 (10)	C9—C8—H8	120.00
C5—C4—C9	117.82 (11)	C8—C9—H9	119.00
C4—C5—C6	121.52 (11)	C4—C9—H9	119.00
C5—C6—C7	119.36 (13)		
			
O2—C1—C2—N1	175.77 (10)	C9—C4—C5—C6	−0.28 (18)
O2—C1—C2—C3	51.79 (15)	C3—C4—C9—C8	−179.15 (11)
O3—C1—C2—N1	−5.77 (16)	C5—C4—C9—C8	0.53 (18)
O3—C1—C2—C3	−129.75 (13)	C4—C5—C6—C7	−0.41 (18)
N1—C2—C3—C4	−57.63 (13)	C5—C6—C7—O1	−178.84 (11)
C1—C2—C3—C4	64.66 (16)	C5—C6—C7—C8	0.88 (18)
C2—C3—C4—C5	94.83 (14)	O1—C7—C8—C9	179.09 (11)
C2—C3—C4—C9	−85.50 (15)	C6—C7—C8—C9	−0.64 (18)
C3—C4—C5—C6	179.40 (11)	C7—C8—C9—C4	−0.07 (19)

### Hydrogen-bond geometry (Å, º)

**Table:**

*D*—H···*A*	*D*—H	H···*A*	*D*···*A*	*D*—H···*A*
N1—H1*N*···Cl1	0.90 (1)	2.36 (1)	3.2326 (12)	162 (1)
N1—H2*N*···Cl1^i^	0.92 (1)	2.44 (1)	3.2872 (12)	154 (1)
N1—H2*N*···O3^i^	0.92 (1)	2.40 (2)	2.9574 (15)	119 (1)
N1—H3*N*···Cl1^ii^	0.90 (1)	2.32 (1)	3.2151 (12)	176 (1)
O1—H1···Cl1^iii^	0.84 (2)	2.36 (2)	3.1858 (11)	169 (1)
O2—H2···O2*W*^i^	0.89 (1)	1.64 (1)	2.5319 (15)	174 (2)
O1*W*—H11*W*···O1^iv^	0.84 (1)	2.10 (1)	2.9044 (13)	162 (2)
O1*W*—H12*W*···Cl1^v^	0.85 (1)	2.33 (1)	3.1784 (11)	172 (2)
O2*W*—H21*W*···O1*W*^i^	0.83 (1)	1.91 (1)	2.7429 (14)	173 (2)
O2*W*—H22*W*···O1*W*^vi^	0.85 (2)	2.02 (2)	2.8318 (15)	161 (2)

Symmetry codes: (i) −*x*+1, −*y*+1, −*z*+1; (ii) *x*+1, *y*, *z*; (iii) −*x*+1, −*y*+1, −*z*+2; (iv) *x*, *y*, *z*−1; (v) −*x*, −*y*+1, −*z*+1; (vi) *x*+1, *y*−1, *z*.
